# Opioid trends in Finland: a register-based nationwide follow-up study

**DOI:** 10.1038/s41598-022-10788-7

**Published:** 2022-05-04

**Authors:** Jaana Keto, Tarja Heiskanen, Katri Hamunen, Maija-Liisa Kalliomäki, Miika Linna

**Affiliations:** 1 Department of Oral and Maxillofacial Disease, Faculty of Medicine, University of Helsinki, Helsinki, Finland; 2 Division of Pain Medicine, Department of Anaesthesiology, Intensive Care and Pain Medicine, University of Helsinki and Helsinki University Hospital, Helsinki, Finland; 3 Department of Anaesthesia, Tampere University Hospital, Tampere, Finland; 4 Department of Health and Social Management, University of Eastern Finland, Kuopio, Finland

**Keywords:** Epidemiology, Pain management

## Abstract

The opioid epidemic in the U.S has gotten payers, prescribers, and policymakers alike interested in trends in opioid use. Despite no recognized opioid crisis in Europe, several countries have reported an increase in opioid-related deaths, which has further prompted discussion on the need of monitoring of opioid prescriptions. This study was conducted to offer information on opioid use during the escalation of the U.S. opioid epidemic in Finland, a Nordic country with universal tax-based health care. This is a nationwide retrospective register-based cohort study on all individuals in Finland who were dispensed opioids in 2009–2017 (n of unique patients = 1,761,584). By using the unique personal identification code assigned to every Finnish resident, we linked data from nationwide registers on dispensed drugs, medical history, and socio-demographic parameters. We report a wide set of patient demographics, dispensing trends for all opioid Anatomical Therapeutic Chemical (ATC) classes, and reasons for opioid initiation based on diagnostic coding for the most recent health care visit. For a cohort of incident opioid users with a four-year wash-out period (n = 1 370 057), we also present opioid use patterns in a three-year follow-up: the likelihood of becoming a persistent user or escalating from weak to strong opioids. A steady 7% of the Finnish population were dispensed opioids annually in 2009-2017. The mean annual quantity of dispensed opioids per opioid patient increased between 2009 and 2017 by 33%, reaching 2 583 oral morphine equivalent mg (OMEQ)/patient/year in 2017. The median quantity of dispensed opioids was lower: 315 OMEQ/year/patient. Depending on the opioid ATC class, there were either increasing or decreasing numbers of patients who had been dispensed said opioid class, and also in the mean quantity. The most common reason for opioid initiation was post-surgical pain (20%), followed by musculoskeletal pain (15%), injury (8.3%), and non-postsurgical dental pain (6.2%). 94% of new opioid initiators started with a weak opioid, i.e. codeine or tramadol. 85% of the patients who had been dispensed a weak opioid were not dispensed an opioid subsequently 3–6 months after the first one, and 95% of them had not escalated to a strong opioid in a 3-year follow-up. The number of patients dispensed opioids in Finland did not change during the escalation of the opioid epidemic in the U.S., but there were changes in the quantity of opioids dispensed per patient. Opioid therapy was typically initiated with weak opioid, the initial dispensed prescription was relatively small, and escalation to strong opioids was rare. A considerable share of patients had been prescribed opioids for chronic non-cancer pain - a type of pain where the risk-benefit ratio of opioids is controversial.

## Introduction

Opioids are effective in managing acute pain and cancer-related pain, but the risk-benefit profile is not as profound for chronic non-cancer pain; adverse effects of opioids range from the very common constipation and nausea to the rare but potentially serious psychiatric depression, addiction, and overdose^1–4^. The number of prescription opioid users in the U.S. reached a peak in the early 2010’s, as did deaths from opioid overdose by prescription opioids or heroin^4–6^. A similar scheme has been seen in Canada, where the number of opioid related deaths has increased from 1993 on^7^. In Europe, there are significant differences in opioid consumption between countries, and there is no continent-wide opioid epidemic comparable to that witnessed in Northern America^1,8–14^. Despite no recognized opioid crisis in Europe, several countries have reported an increase in opioid-related deaths, which has further prompted discussion on the need of monitoring of opioid prescriptions^1,11–14^.

We set out to inspect opioid trends in Finland, a country of some 5.6 million inhabitants and universal tax-funded health care, during the escalation of the opioid epidemic in the U.S. As a strong association has been established between patient demographics and the probability of misuse and other adverse events, we wanted to include also patient demographics in our study^1,12,15^.

The objectives of this study were to inspect patient characteristics and trends in opioid use over 8 years, to unveil indications for initiating opioid therapy in the total population, and to present typical patterns of opioid use in a two-year follow-up after initiation of opioid therapy.

## Methods

### Study design

This study was conducted as a nationwide retrospective register-based cohort study using data obtained from the Finnish social and health care registers, which are regularly audited for completeness and accuracy^16^. Finnish nationwide registers contain individual-level data on a broad range of variables, such as diagnoses, medication, mortality, health care resource use, treatment costs, as well as sociodemographic and socioeconomic characteristics for the total population of 5.6 million inhabitants. Data from these administrational registers can be linked with the unique personal identification number, which allows the investigator to follow the journey of the patient in the social and health care system^17^.

### Setting

The study population consisted of all patients in Finland who were dispensed opioids during 2009–2017. The patients were followed up until Dec 2017. The data was collected retrospectively from the nationwide registers. The study was carried out in accordance with the national regulations, and the study permits were granted by the state agencies who act as data owners, as listed in Table 1. The data owners pseudonymised the data, i.e. replaced personal identification numbers (PIN) used in linking data from the different registers with subject identification numbers (SID) prior to handing the data to the investigators. According to the national regulations, informed consent is not needed for studies based on pseudonymised secondary data. Collecting consent would also not be possible, as the investigators do not know the identities of the subjects.

**Table 1:** Data sources and information obtained.

Data source	Register holder	Information obtained
National prescription register	The Social Insurance Institution of Finland	Dispensed opioids reimbursed by the state
Register on long-term sick leave	The Social Insurance Institution of Finland	Decision on long-term sick leaves
Reimbursement register	The Social Insurance Institution of Finland	Decisions for reimbursement of private sector health care use
Care register for secondary and primary health care	National Institute for Health and Welfare	Diagnoses (ICD-10 codes) and interventions
Cause of death register	Statistics Finland	Deaths and causes of deaths
Register of completed education and degrees	Statistics Finland	Education level
Population register	Population Register Center	Date of birth and place of domicile

### Participants

All Finnish residents who had at least one opioid dispensing event in the national prescription register during 1st January 2009–31st December 2017 were included in the study cohort. Each patient was included in the study cohort only once. The patient’s index date was defined as the date of first time of being dispensed an opioid in at least four years. Data on treatment and co-morbidity history were gathered to cover at least four years of the patient’s medical history prior to the index date, from the period 1st January 2005–31st December 2017.

Follow-up data on primary and secondary health care use was gathered for all patients after their index date. The follow-up ended either on 31st December 2017, at time of death, or at time of emigration, whichever occurred first.

### Variables

#### Opioid use

Trends in opioid use during 2009–2017 are reported for each calendar year as the number of unique patients who were dispensed opioids, and as the quantity of opioids dispensed. Opioid quantity is expressed as oral morphine equivalents in milligrams (OMEQ mg).

In 2009–2017, opioid use in Finland consisted of codeine combinations, tramadol, and dextropropoxyphene which are classified as weak opioids in the WHO analgesic ladder, and of oxycodone, morphine, hydromorphone, fentanyl, pethidine, and transdermal and sublingual buprenorphine, which are classified as strong opioids. We report opioid use for each opioid ATC class separately, and as the total sum of all opioid classes. Pethidine was not included in the analyses, as it had been dispensed to only four patients under a special license. Dextropropoxyphene was not included in the analyses, as it was available only until 2010.

#### Opioid patient characteristics

For the cohort of all Finnish opioid patients in 2009–2017, the following characteristics are reported: sex, age, education level (basic, intermediate, high), cancer status before and after initiation of opioid therapy, residency in a large city, postsurgical patients, psychiatric patients, multimorbid patients (patients with at least two chronic diseases), patients with prior long sick leave, and the size of the first dispensed opioid prescription in OMEQ mg.

#### Indication for opioid initiation

The likely indication for initiation of opioid therapy was explored by inspecting diagnostic and administrative coding for the nearest health care visit prior to the opioid dispensing event. The indications were grouped based on the ICD-10 chapter the patient’s diagnosis fell under, for instance Cancer pain was ICD-10 chapter C, Injury was ICD-10 chapter S, Musculoskeletal pain was ICD-10 chapter M. The full ICD-10 classification can be found at https://www.who.int/standards/classifications/classification-of-diseases. As an exception, dental patients were defined as patients whose most recent health care service had been provided by a dentist, or where the ICD-10 code had been K00-K14. Postsurgical patients were defined as patients who had initiated an opioid within one week of discharge date after surgery, based on Nordic Classification of Surgical Procedures operating room procedure coding.

#### Opioid use patterns

The probability of becoming a persistent user, or progressing from weak (codeine combinations, or tramadol) to strong opioids (oxycodone, morphine, hydromorphone, fentanyl, or buprenorphine) are reported in 3-year follow-up for patients who initiated opioid therapy in 2009-2014 (n = 1 370 057). The patients had a four-year wash-out period during which they could not have been dispensed opioids. Persistent use was defined as being dispensed more opioids during months 3-6 after the first dispensed prescription.

### Data sources

The data sources for all variables are listed in Table 1.

#### Quantity of dispensed opioids

OMEQ is, from a clinical point of view, the preferred measure of opioids, as it accounts for differences in analgetic efficacy between different opioids and their means of administration^18^. This parameter does not appear in the Finnish National prescription register from which we collected information on dispensed opioids. The register should, however, carry the Defined Daily Dose (DDD) value for each dispensed prescription, which could be converted to OMEQ. When we reviewed the data delivered to us, we noticed that only a fraction of the unique dispensing events carried the DDD information. As such level of data completeness is not sufficient for research purposes, we calculated the OMEQ for each opioid sales article ourselves. We did this by using the unique sales article code (Vnr) associated with each opioid dispensing event. All pharmaceutical products sold in Finland have a unique sales article code, which carries information about the active ingredient, dose strength, formulation, and package size for each sales article. From this information, we calculated the OMEQ for each opioid sales article that had been available in Finland during our study period, and for each opioid dispensing event. In the U.S., the FDA has facilitated opioid research by offering this information on opioid sales articles to investigators in a so called Opioid Analytical File^19^ The Finnish Opioid Analytical File created by us for the present study is available for other investigators by request.

### Bias

This is an unselected national cohort of all patients in Finland who had been dispensed opioids during 2009–2017 according to the National prescription register. All Finnish residents are covered by national health insurance, which includes reimbursement of prescription medications, such as opioids, leaving the national prescription register with a practically full coverage of the national population.

### Statistical methods

We report patient characteristics for the full cohort of Finnish residents who were dispensed opioids in 2009–2017 in absolute numbers, means and percentages, as applicable.

For the number of patients dispensed opioids each year, we report absolute values (n) and n/100,000 inhabitants. The denominator—the population of Finland on Dec 31 of each calendar year—was retrieved from the official population statistics of Finland (http://www.stat.fi/til/vaerak/index.html). We also report the mean and median quantity of opioids dispensed per opioid patient for each calendar year.

For each opioid ATC class, we calculated the mean quantity dispensed each year a) per patient, b) per 1,000 patients, and c) per 1,000 inhabitants.

For the indication of opioid initiation, we report percentages of the total cohort of opioid users during 2009-2017.

We present opioid use patterns in two-year follow-up in a Sankey diagram. In the diagram, we first report percentages of patients who initiated opioid therapy with a weak or a strong opioid, in relation to the cohort of patients who initiated opioid therapy in 2009-2014 (n = 1 370 057). We then report the respective percentages of persistent, non-persistent, or dead patients at six months after opioid initiation. We continue to report the percentages of patients who had or had not progressed to or continued using strong opioids, and who were dead at 36 months after opioid initiation.

Stata version 15.0 was used for all analyses (StataCorp. 2017).

### Ethics approval and consent to participate

Not applicable based on Finnish national legislation on secondary use of pseudonymised health data.

## Results

### Opioid patient characteristics

The study cohort consisted of the 1,761,584 unique patients who were dispensed an opioid at least once during 2009-2017, with a mean age of 52.5 years. The mean size of the first dispensed opioid prescription was 256 OMEQ mg. The patient baseline characteristics for the cohort are presented in detail in Table 2.

**Table 2:** Baseline characteristics at initiation of opioid therapy for all opioid users in Finland in 2009–2017 (n = 1 761 584).

	Women	Men	All
N	930,120	831,464	1,761,584
Mean age	53.9	50.9	52.5
Age <18 (n)	1.6% (15,148)	1.6% (13,566)	1.6% (28,714)
Age 18-75 (n)	79.9% (743,392)	87.5% (727,365)	83.5% (1,470,757)
Age >75 (n)	18.4% (171,580	10.9% (90,533)	14.9% (262,113)
Basic education (n)	30.9% (287,285)	29.9% (248,338)	30.4% (535,624)
Inter med education (n)	37.9% (352,732)	44.0% (366,088)	40.8% (718,820)
High education (n)	30.7% (285,309)	25.3% (210,185)	28.1% (495,495)
Resident in large city (n)	24.5% (227,663)	22.7% (188,798)	23.6% (416,461)
Multimorbid (n)	66.2% (615,595)	54.4% (452,354)	60.6% (1,067,950)
Psychiatric patient (n)	14.2% (131,917)	11.1% (92,028)	12.7% (223,945)
Prior cancer diagnosis (n)	8.4% (78,540)	8.7% (72,616)	8.6% (151,156)
Cancer diagnosis after opioid initiation (n)	10.7% (99,675)	11.9% (98,578)	11.3% (198,252)
Prior long sick leave (n)	9.4% (87,767)	10.1% (83,854)	9.7% (171,621)
Mean size of initial dispensed opioid prescription (OMEQ mg)	252.9	259.6	256.1

### Opioid use

**Figure 1:**
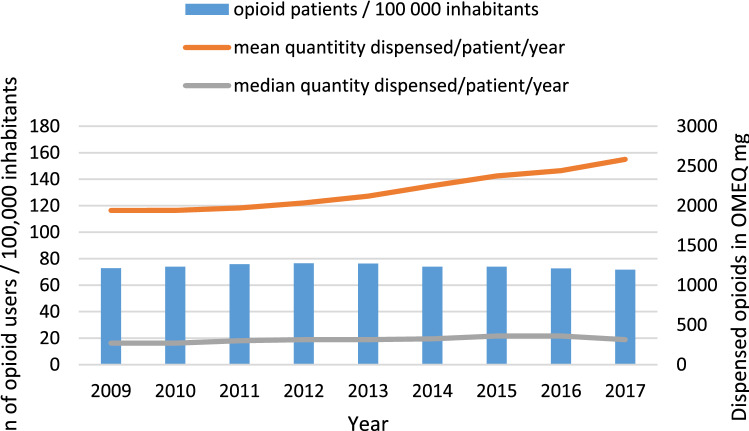
Number of patients who had been dispensed opioids per 100,000 inhabitants, and the mean and median quantity of opioids dispensed per patient per year in oral morphine equivalents (OMEQ mg) in 2009-2017.

The number of individual patients who were dispensed opioids in relation to 100,000 inhabitants remained stable during the nine-year inspection period (Fig. 1). Each year some 400,000 (range 389,526-415,00) patients—7% of the population of Finland—were dispensed opioids.

The stable sized patient pool used opioids in increasing intensity. The median quantity of opioids dispensed per patient per year ranged between 270 and 360 OMEQ mg/patient/year during 2009-2017. The increase in the median annual quantity per patient was 17% during 2009-2017. The mean was substantially higher than the median, ranging between 1,940 OMEQ and 2,583 OMEQ mg/patient/year (Fig. 1), with a 33% increase during 2009-2017. The mean quantity of dispensed opioids in relation to the population was 185,157 OMEQ mg/1,000 inhabitants/year in 2017.

**Figure 2:**
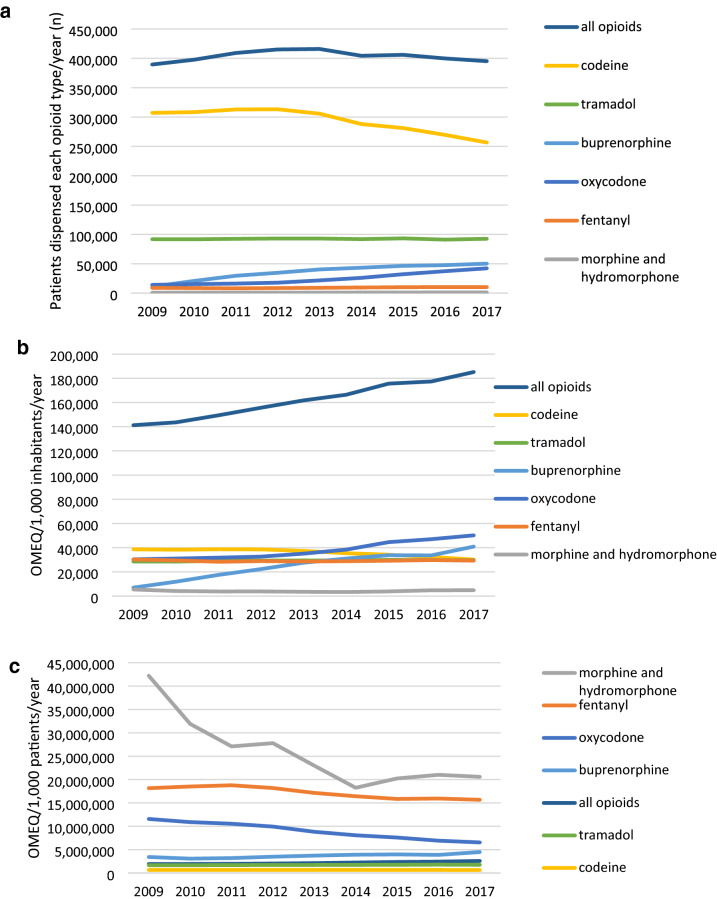
(**a)** Number of patients with a dispensed opioid prescription during the calendar year, reported by opioid ATC class. (**b**) Opioid use in oral morphine equivalent mg (OMEQ) per 1,000 inhabitants in Finland, reported by opioid ATC class. (**c**) Mean intensity of opioid use per 1,000 opioid patients by opioid class in Finland. Intensity is expressed in oral morphine equivalent mg (OMEQ) for 1,000 patients who used each opioid class during the calendar year.

For weak opioids, the use of codeine combinations decreased during 2009-2017 in terms of patient numbers and dispensed quantity per 1,000 inhabitants, with a relatively unchanged dispensed quantity per patient (Fig. 2a–c).

For strong opioids, out-patient use of fentanyl and (hydro)morphine remained stable during the inspection period of 2009-2017, with a decreasing mean dispensed quantity per patient for both opioid classes. While the patient numbers for both buprenorphine and oxycodone increased, the mean amounts dispensed took opposite trends for these two opioids: buprenorphine saw an increase, oxycodone a decrease in dispensed quantity per patient.

### Indication for opioid initiation

**Figure 3:**
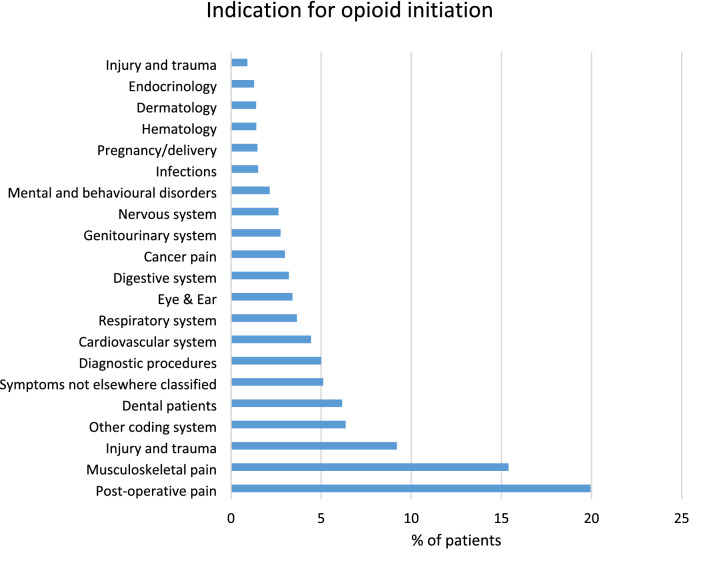
Indication for opioid initiation based on diagnostic coding for the most recent health care visit prior to the first dispensed opioid prescription. All percentages in the figure are proportions of the total patient cohort of 1,761,584 opioid users.

Postsurgical pain was the most common indication for initiating opioids (19.9%), followed by musculoskeletal pain (15.4%), injury (8.3%), and non-postsurgical dental pain (6.2%) (Fig. 3). Of the whole opioid patient population, 3% had initiated opioids for cancer pain (Fig. 3).

### Opioid use patterns

**Figure 4:**
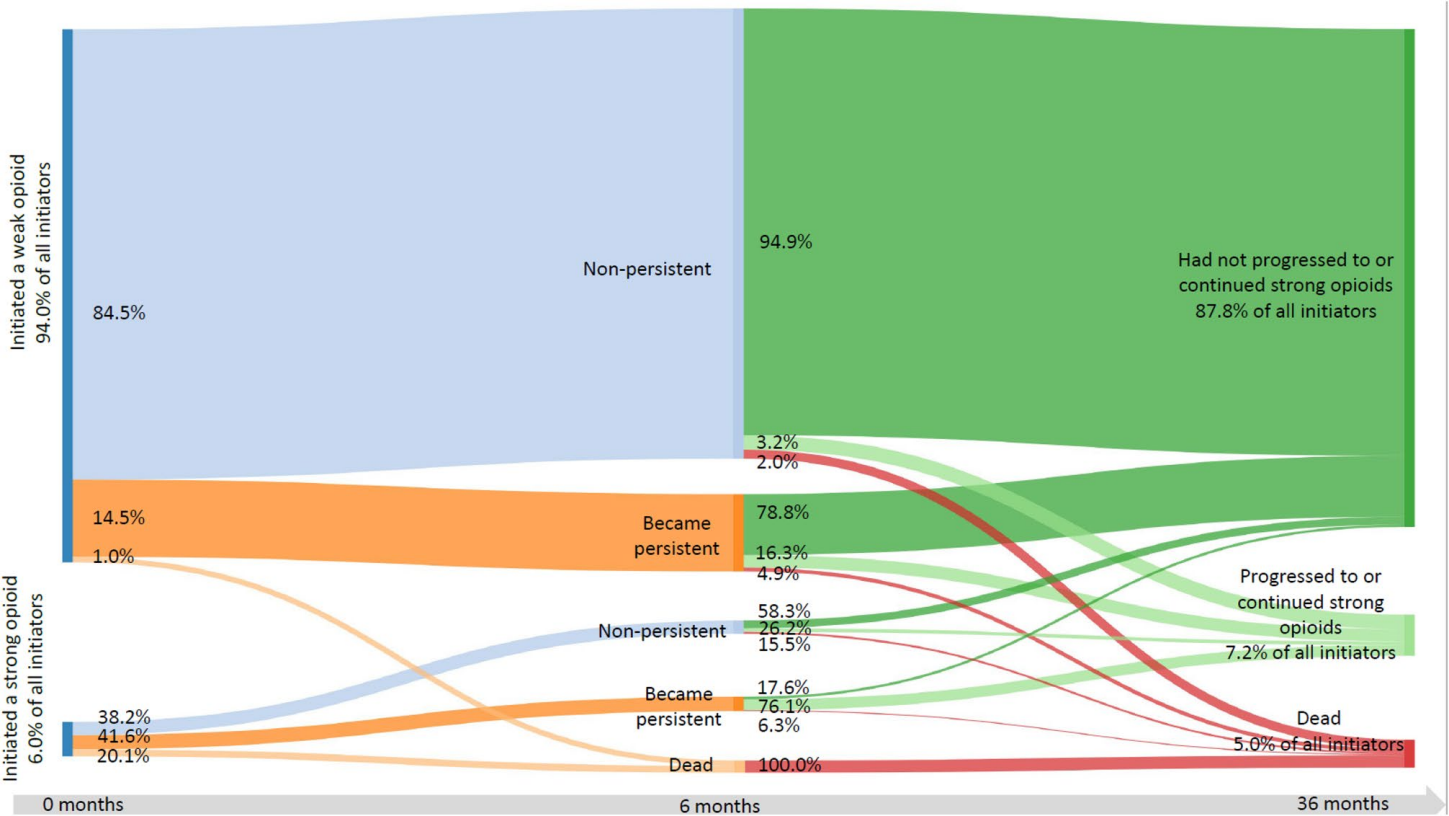
Patterns of opioid use in three-year follow-up for patients who initiated opioid therapy in 2009-2014 (n = 1,370,057). Persistent use was defined as more opioids dispensed between months 3-6 after the initial dispensed prescription.

Paths to becoming a persistent user, or to using strong opioids are presented for patients who initiated opioid therapy in 2009-2014 (n = 1,370,057) in Fig. 4. Of all opioid initiators, 94.0% started their opioid treatment with a weak opioid. 14.5% of the patients who started with a weak opioid became persistent users, compared with 41.6% of those who had initiated their opioid therapy with a strong opioid. 5.0% of patients who had initiated treatment with a weak opioid were dispensed a strong opioid within three years of opioid initiation (Fig. 4).

## Discussion

In this study, we report patient characteristics and trends in opioid use over 9 years in 2009-2017, unveil reasons for initiating opioid therapy in the total population, and present typical patterns of opioid use in a two-year follow-up after initiation of opioid therapy. The inspected time period coincides with the escalation of the opioid epidemic in the United States^3,5,6^.

Annually, some 7% of the Finnish population were dispensed opioids for the treatment of pain, and no increase in number of patients using opioids was seen over the period of nine years. A previous analysis on the Finnish working-age population also reported a flat trend in the number of working-age opioid users^20^. The stable-sized pool of patients were dispensed increasing quantities of opioids per patient, reaching a mean of 2 583 OMEQ mg per patient per year. In Northern European comparison, this was on the low end: opioid use in Scandinavia varies between the 2 426 OMEQ mg dispensed on average per patient/year in Norway and the 6 361 mg/patient/year in Denmark in 2014^21^. The median quantity of dispensed opioids was much lower than the mean - 315 OMEQ/patient/year. This corresponds to, for instance, 90 tablets of a 30 mg codeine / 600 mg paracetamol combination product, the most commonly sold opioid product in Finland. The median quantity of dispensed opioids was also close to the mean size of the initial dispensed prescription, 256 OMEQ/patient.

While unselected nationwide opioid patient databases are rare outside the Nordic setting, drug sales statistics allow inspection of opioid sales in relation to population size. With such methodology, Bosetti et al. found that after a steady increase of sales of selected strong opioids in most European countries in the 1990s, the sales levelled off or even declined in some countries in the 2010s^9^. A slightly decreasing trend has also been reported in the United Kingdom for 2015–2018^14^. However, there have been also opposite reports from Netherlands, France, and Germany, where the investigators have also been able to link opioid prescriptions with clinical data^11–13^.

To visualise the added value of inspecting patient-level data over national sales statistics, we have reported opioid use in OMEQ both in relation to 1,000 inhabitants, and to the number of patients who had been dispensed each opioid class during the given year. This revealed interesting trends: For oxycodone, the amount dispensed annually per 1,000 inhabitants rose by 65%, but patient-level data revealed that the mean dispensed quantity per patient decreased by 43%. For codeine combinations, the quantity dispensed per 1,000 inhabitants declined by 22%, which was due to a 16% decrease in the number of patients dispensed codeine for pain, with the mean quantity dispensed per patient remaining nearly unchanged. Including patient demographics in the analyses revealed to which extent opioids were dispensed to patients belonging to established risk groups for opioid-related adverse events, such as misuse^15^.

Inspection of opioid trends on a detailed level is crucial if one wishes to assess the effectiveness of opioid risk management measures that have already been taken, or to assess the need and target for future measures. In Finland, the current control measures for strong opioids include the need for a special prescription, and a three-month time limit for national reimbursement, after which a new prescription needs to be made. The prescription of the weakest available opioid, codeine, has also been subject to control measures: the authorities have identified heavy prescribers of codeine from the national prescription database, and approached them with targeted education letters. Ahomäki et al. have recently modelled the effect of such a targeted letter sent by national authorities to high prescribers of codeine combinations^22^. They estimated that the average number of codeine combination tablets dispensed to new patients decreased by 12.5 percent as a result of the targeted letter. We would welcome a similar analysis for the impact of the letter on opioid prescriptions across all opioid classes, measured in OMEQ, to see if the high prescribers have shifted from codeine combinations to stronger opioid classes. Furthermore, it would be interesting to see which patient groups’ access to opioids was impacted by the letter: young patients with psychiatric disorders, or middle-aged patients with post-operative pain from a recent operation, for instance.

The present study revealed that approximately one third of all opioid initiators most likely suffered from acute pain: they were either postoperative patients, cancer patients, dental patients, or had recently suffered an injury which needed medical attendance. The remaining proportion of the patients was heterogeneous in terms of morbidity. Diseases of the musculoskeletal system were common, with a patient share of 15%, similar to what has been reported in Ontario, Canada^23^.

There is no single agreed-upon definition of persistent opioid use, and the definition also depends on the nature of data available to the investigators^24^. With our rather inclusive definition, i.e., having more opioids dispensed within 3 to 6 months from the first one, the proportion of persistent users—15%—was very low compared to a national database study from the United Kingdom, where every other patient using an opioid for non-cancer pain was estimated to have been prescribed an opioid continuously for at least 12 months^14^. In Finland, almost all (94 %) patients initiate their opioid therapy with a weak opioid, and the initial dispensed prescription consists, on average, of 256 OMEQ, which corresponds approximately to a package of 50 tablets of a codeine combination. The small percentage of patients initiating opioid therapy with a strong opioid had a higher risk of persistent opioid use or death when compared with patients whose initial opioid prescription was for a weak opioid. Adjusting for baseline differences in age or morbidity status could produce different results.

### Strengths and limitations

As a strength, this study has minimal selection bias: it relies on the dispensed opioid prescriptions and linked health history of all inhabitants in Finland, regardless of whether they were treated in the private or the public sector, in primary or secondary care. As a limitation, there is no knowing whether the patient consumed the dispensed opioid or not. Our data set also does not include illicit opioid use, or opioid use that took place in a hospital setting. Furthermore, the study has highest relevance to other countries with tax-funded public health care, and limited generalisability for countries with insurance-based health care system.

## Data Availability

The data that support the findings of this study are available from the Finnish Social and Health Data Permit Authority Findata by request.

## Abbreviations

OMEQ: Oral morphine equivalent milligrams
DDD: Defined daily dose
FDA: United states food and drug administration
U.S.: United States of America
ATC: The anatomical therapeutic chemical classification system of pharmaceuticals
